# Successful Early Thrombolysis of Anterior ST-Elevation Myocardial Infarction (STEMI) in a COVID-19 Patient: A Case Report

**DOI:** 10.7759/cureus.93871

**Published:** 2025-10-05

**Authors:** Dereck A Kaale, Filemon Mmary, Nicole Kinyawa, Deus N Kitapondya, Elizabeth E Mmari

**Affiliations:** 1 Emergency Medicine, Université Grenoble Alpes, Grenoble, FRA; 2 Internal Medicine, Saifee Hospital Tanzania, Dar es Salaam, TZA; 3 Critical Care Medicine, Jakaya Kikwete Cardiac Institute, Dar es Salaam, TZA; 4 Emergency Department, Ephata Mission Hospital, Tabora, TZA; 5 General Surgery, Saifee Hospital Tanzania, Dar es Salaam, TZA

**Keywords:** acute myocardial infarction, covid-19, fibrinolysis, microvascular occlusion, thrombolysis

## Abstract

Coronavirus disease-2019 (COVID-19) primarily affects the respiratory system, but its complications have been seen in other systems, including the cardiovascular system. Chest pain is one of the most common complaints in the individuals affected with COVID-19 disease and has to be thoroughly investigated. It might be associated with fatal pathologies like acute myocardial infarction (AMI). This case demonstrates an eventual diagnosis and treatment of AMI in a patient admitted for COVID-19 disease.

## Introduction

In March 2020, the World Health Organization (WHO) declared COVID-19 to be a pandemic. Since then, the disease has been changing its pattern of presentation. The change in patterns has created a global healthcare burden, associated with high mortality and morbidity rates all over the world [1].

Despite the primary target being the respiratory system, the cardiovascular system is also one of the most affected systems by the coronavirus, either directly or indirectly, resulting from inflammation, endothelial activation, and microvascular thrombosis that arises in the context of the disease [2].

COVID-19 can cause myocardial infarction by triggering widespread inflammation, endothelial damage, and a hypercoagulable state that promotes plaque rupture and clot formation in coronary arteries. It also worsens oxygen supply-demand mismatch through hypoxia and direct cardiac injury. Both vascular spasms, microvascular thrombosis, and hypoxia can lead to acute myocardial infarction (AMI) in patients with COVID-19 disease [3].

We hereby present a case of a 56-year-old woman who was admitted for COVID-19 treatment and developed AMI in the ward.

## Case presentation

Patient information

A 56-year-old woman presented to the emergency department (ED) with a one-week history of progressive general body weakness and a day history of shortness of breath. She is a known patient with hypertension, type 2 diabetes mellitus, and gout on Losartan, aspirin, metformin/glibenclamide, allopurinol, and meloxicam.

Clinical findings

On arrival, she was alert, oriented, dyspnoeic, and diaphoretic with the following vital signs: blood pressure 174/107 mmHg, heart rate 89 beats per minute, respiratory rate 18 cycles per minute, oxygen saturation 88% in room air, and temperature 37°C. She was kept on oxygen support at a rate of 15 L/min via a non-rebreather mask, and her oxygen saturation increased to 99%.

Diagnostic assessments

**Figure 1:**
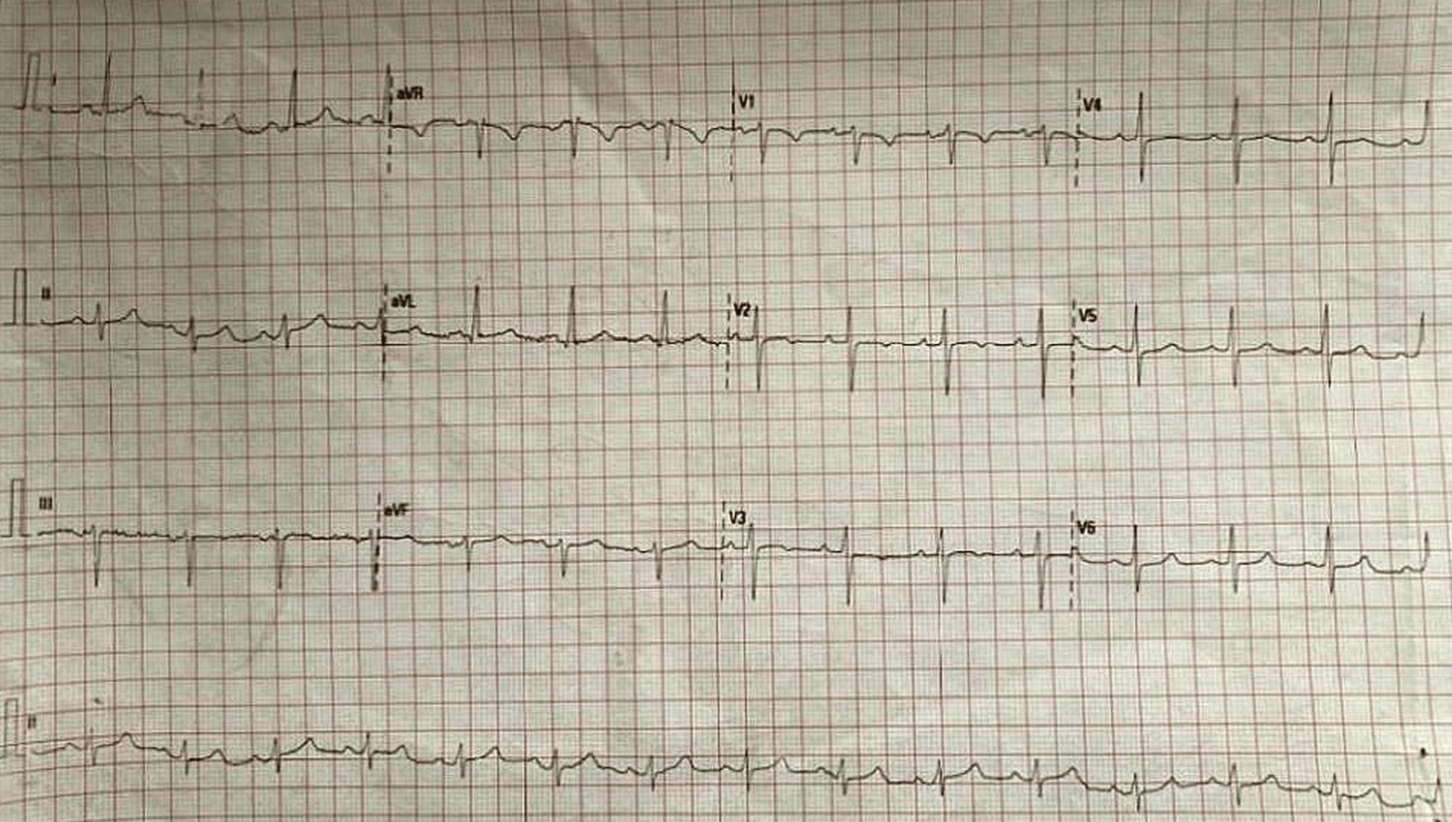
Normal sinus rhythm 12-lead ECG

A 12-lead electrocardiogram (ECG) was performed (Figure 1) and revealed a normal sinus rhythm.

A chest computed tomography scan (CT scan) was performed before arrival in the ED and revealed mediastinal adenopathy with an extensive bilateral atypical pneumonia with a CT severity score of 23/25. Subsequent workup with a complete blood count was normal. C-reactive protein was 113.5 mg/L, D-dimer was 14.76 ng/ml, and within-limit arterial blood gases (ABG), which showed that respiratory alkalosis, prothrombin time/international normalized ratio (PT/INR) and partial thromboplastin time (PTT) were within normal limits. Two-dimensional echo-cardiogram (2D ECHO) showed an ejection fraction (EF) of 67%. RT-PCR test was positive for COVID-19. The patient was admitted to the isolation ward and started on COVID-19 treatment, which included antiviral medications, steroids, and low-molecular-weight heparin.

Therapeutic interventions

**Figure 2:**
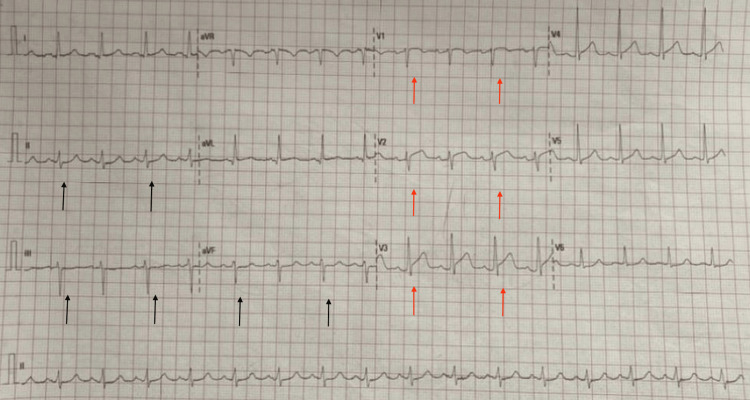
12-lead ECG showing ST segment elevation (STE) in V1 through V3 (red arrows) with reciprocal changes in the inferior leads (black arrows)

The next day after admission, the patient started experiencing chest pain. A 12-lead ECG was done (Figure 2) and revealed ST-segment elevation (STE) in V1 through V3 with reciprocal changes in the inferior leads correlating with anterior wall MI.

She was immediately loaded with aspirin, clopidogrel, and atorvastatin tablets and planned for immediate thrombolysis with alteplase.

**Figure 3:**
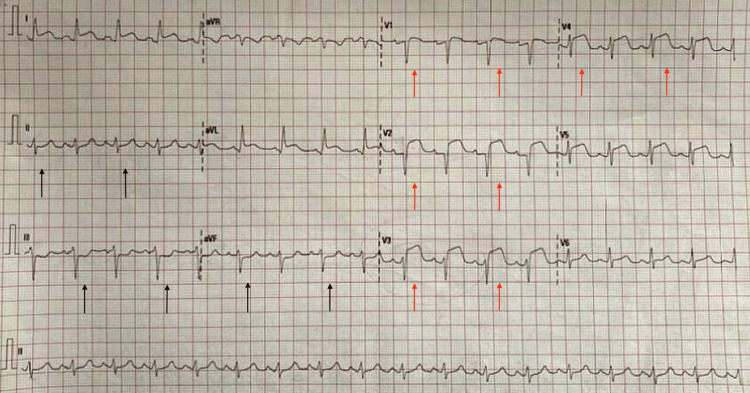
12-lead ECG before thrombolysis with prominent ST segment elevations (STE) on the anterior leads (red arrows) and reciprocal changes in inferior leads (black arrows)

Troponin I was elevated and was >50 ng/mL. Consent was obtained from the family, and a second 12-lead ECG (Figure 3) was performed immediately before thrombolysis, revealing ST segment elevation (STE).

**Figure 4:**
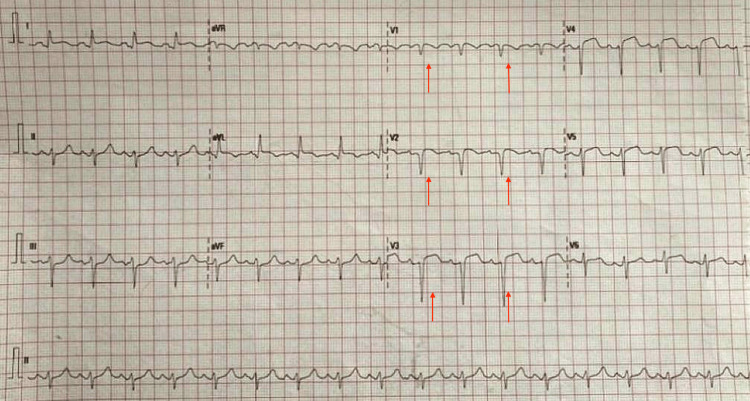
12-lead ECG obtained one hour after thrombolysis showing more than 50% resolution of the ST segment elevation (STE) on the anterior leads (red arrows)

The patient was then thrombolyzed within one hour of symptom onset using alteplase 15 mg intravenous (I.V.) bolus over two minutes followed by 0.75 mg/kg I.V. infusion for half an hour, and then 0.5 mg/kg I.V. over the next hour. A 12-lead ECG (Figure 4) was obtained one hour after thrombolysis and showed ST-segment resolution by more than 50%, associated with clinical resolution of chest pain.

Follow-up and outcomes

The 2D ECHO showed apex and anterior wall hypokinesia with an EF of 46%. Follow-up ECGs, troponin I, and 2D ECHO were done every 12 to 24 hours. The patient was constantly monitored with daily significant improvements clinically and investigation-wise associated with oxygen weaning off. On day 8, she was discharged home.

## Discussion

AMI has been observed in up to 62% of patients with COVID-19 disease, with higher incidents of mortality in those with severe infection or comorbidities, as in our case. Shock and arrhythmia are being reported to be the most common outcomes of acute cardiac injury [4]. The diagnosis of AMI should be confirmed through a combination of history, high-sensitivity troponin, and imaging modalities, including 12-lead ECG, echocardiography, cardiac magnetic resonance imaging (MRI), or cardiac CT scan [4,5]. Primary percutaneous coronary intervention (PPCI) remains the gold standard of care for treating COVID-19 patients with ST-elevation myocardial infarction (STEMI), as guideline recommendations were reached after consensus from different colleges, societies, and associations of cardiologists globally [4-6].

In scenarios where PPCI is not possible, thrombolysis should be considered early and administered promptly in the absence of contraindications, with the greatest value being within one hour of pain onset [6,7].

## Conclusions

This case highlights the importance of a thorough cardiovascular examination in all individuals diagnosed or suspected of having COVID-19. Different cardiologist societies, colleges, and associations still recommend PPCI as the gold standard for AMI treatment. In the absence of or contraindication to PPCI, early thrombolysis within the first hour of AMI diagnosis may reduce in-hospital mortality in patients undergoing COVID-19 treatment.
